# Circulating plasma cells: from basic mechanisms to clinical applications

**DOI:** 10.3389/fimmu.2025.1674088

**Published:** 2025-10-30

**Authors:** Pingping Wang, Nan Su, Xiaojing Yan, Feng Xu, Yan Zhang

**Affiliations:** Department of Hematology, The First Hospital of China Medical University, Shenyang, China

**Keywords:** plasma cells, circulating plasma cells, plasma cell disorders, multiple myeloma, flow cytometry

## Abstract

Circulating plasma cells (CPCs) represent an accessible subset of antibody-secreting cells that provide valuable insights into immune activation and regulation. This review presents the first comprehensive synthesis of CPC biology, with a particular focus on the mechanisms governing their generation under physiological conditions and the distinct pathways that drive their formation within tumor microenvironments. We further summarize the broader clinical relevance of CPCs as potential biomarkers across infections, autoimmune diseases, and plasma cell disorders. With the rapid advancement of liquid biopsy technologies, CPC detection has garnered increasing attention in clinical practice. Here, we evaluate current and emerging methods for CPC detection, highlighting their respective advantages and limitations. Finally, we discuss the translational potential of CPCs and outline future research directions to support more precise diagnosis and treatment strategies in CPC-associated conditions.

## 1 Introduction

Plasma cells, the terminal effectors of the B-cell lineage, are key effector cells in humoral immunity through their production of antibodies and maintenance of immune memory. While most plasma cells reside in specialized niches such as bone marrow, spleen, and mucosa-associated lymphoid tissues, a small population of circulating plasma cells (CPCs) persists in peripheral blood (1). Traditionally considered short-lived and rare, CPCs were thought to reflect routine immune turnover under steady-state conditions, often representing a transitory state between plasmablasts, long-lived plasma cells (LLPCs), or apoptotic cells, particularly in non-disease states. However, CPC populations fluctuate significantly in response to infections, autoimmune diseases, and plasma cell disorders. Recent evidence demonstrates that CPCs expand following vaccination and infection, implicating their involvement in systemic immune surveillance and adaptive memory formation (2, 3). In autoimmune diseases such as systemic lupus erythematosus (SLE), elevated CPC levels correlate with autoantibody production and chronic inflammation, highlighting their potential as dynamic biomarkers for disease activity and therapeutic response (4). In plasma cell disorders, particularly multiple myeloma (MM), CPCs are associated with tumor burden and have shown promise as minimally invasive indicators for early screening and diagnosis, prognostic evaluation, response assessment and therapeutic guidance (5, 6). In MM, CPCs typically reflect clonal plasma cells that have exited bone marrow due to niche remodeling, rather than newly formed CPCs from B-cell activation. Although plasma cell biology has been extensively studied, most existing reviews have concentrated on LLPCs or precursor states rather than CPCs. For instance, Nguyen et al. summarized intrinsic programs such as apoptosis resistance, autophagy, and metabolic regulation, together with extrinsic factors like bone marrow stromal support and cytokine signaling, as key determinants of LLPC survival (7). Manakkat Vijay & Singh synthesized recent insights into the temporal dynamics of generation of plasma cell precursors during germinal center responses (8). While these contributions have advanced our understanding of plasma cell maturation, the biology of CPCs remains insufficiently defined. In particular, the mechanisms that regulate CPC generation under physiological conditions, as opposed to those driving their aberrant expansion within tumor-associated microenvironments, are still unclear. This gap in knowledge has hindered the translation of CPC biology into clinical practice, despite their considerable potential to serve as minimally invasive biomarkers across diverse disease settings.

In this review, we present the first comprehensive synthesis of CPC biology, systematically detailing the mechanisms underlying CPC generation under both physiological conditions and tumor-associated microenvironments, offering an integrated perspective that extends beyond the scope of previous plasma cell-focused reviews. We also highlight recent advances in CPCs research and broaden their clinical relevance beyond MM to include autoimmune diseases, emphasizing their emerging value as biomarkers at the interface of immunology, hematology, and oncology. Finally, we examine current and next-generation CPC detection technologies, and discuss their translational potential in precision medicine, along with future directions for basic and clinical research.

## 2 Biological characteristics of normal plasma cells

### 2.1 Origin and development

B cells differentiate into plasmablasts, which are precursors of both short-lived and LLPCs; the latter represent the terminal stage of B cell differentiation. This transition is accompanied by extensive transcriptional reprogramming and has been viewed as a shift from a proliferative plasmablast stage to a non-dividing, antibody-secreting plasma cell state. Notably, recent findings indicate that only a small fraction (~10%) of circulating human plasmablasts are proliferative, while most have already exited the cell cycle (9). Following their generation in the lymph nodes, plasmablasts transiently circulate and subsequently home to specific microenvironments like the bone marrow, spleen, mucosa-associated lymphoid tissue (MALT), or lymph nodes. These microenvironments constitute niches that provide essential survival signals to early-stage plasma cells in peripheral blood.

**Figure 1:**
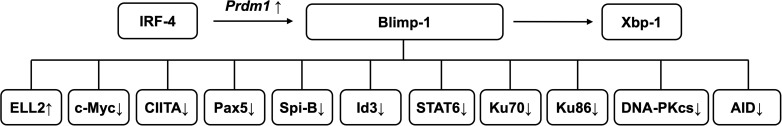
The critical drivers of plasma cell generation. The transcription factors Blimp-1, Xbp-1, and IRF-4 are critical regulators of B cell differentiation into plasma cells. *IRF-4* indirectly promotes plasma cell development by up-regulating *Prdm1* transcription, leading to increased *Blimp-1* expression. In turn, *Blimp-1* drives plasma cell differentiation by suppressing the expression of genes involved in B cell receptor signaling (*Pax5*, *Spi-B*, *Id3*, *STAT6*), germinal center B cell function (*Ku70*, *Ku86*, *DNA-PKcs*, *AID*), proliferation (*c-Myc*), and MHC-II presentation (*CIITA*). Concurrently, Blimp-1 promotes the expression of genes essential for plasma cell identity, such as *Xbp-1* and *ELL2*. *Xbp-1* further modulates plasma cell differentiation through the unfolded protein response (UPR).

In general, plasma cell differentiation initiates with the activation of B cells in secondary lymphoid organs, typically characterized by a reprogramming of transcriptional networks: the down-regulation of B cell-promoting factors like *Pax5*, *Bach2*, and *Bcl6*, and the up-regulation of plasma cell-promoting factors including *Blimp-1*, *Xbp-1*, and *IRF-4* (10–13). *Blimp-1*, *Xbp-1* and *IRF-4* are considered the pivotal drivers of plasma cell development (**Figure 1**). Throughout the activation and directed differentiation of B cells into plasma cells, the suppression of *Pax5* expression relieves its inhibitory effect on the plasma cell transcriptional program, thereby accelerating the expression of *Blimp-1* and *Xbp-1* (10). *Blimp-1* predominantly drives plasma cell differentiation by suppressing *c-Myc*, *class II transactivator (CIITA)*, *Pax5*, and B-cell receptor signaling components (e.g., *Spi-B*, *Id3*), inhibiting immunoglobulin class-switch recombination by down-regulating activation-induced cytidine deaminase (*AID*), *Ku70*, *Ku86*, DNA-dependent protein kinase catalytic subunit (*DNA-PKcs)*, and signal transducer and activator of transcription 6 (*STAT6)*, strengthening the expression of pro-immunoglobulin secretion genes such as *ELL2*, while permitting the expression of crucial plasma cell genes like *Xbp-1* (14–17). Meanwhile, Blimp1 bound to and activated 20 genes coding for proteins implicated in ER function and ER stress control in plasmablasts, directly contributing to the regulation of immunoglobulin secretion (17). *IRF-4* promotes plasma cell development by enhancing *Prdm1* transcription (encoding for *Blimp-1*), leading to elevated Blimp-1 expression (18). As a downstream effector of *Blimp-1*, *Xbp-1* orchestrates plasma cell function and immunoglobulin secretion through the unfolded protein response (UPR) (19). Genes like *ERdj3* and *OBF-1* have been identified as direct *Xbp-1* targets involved in both plasma cell differentiation and classical UPR pathways (19). *Xbp-1* also regulates transcription of the immunoglobulin heavy chain by controlling heavy chain–specific transcription factors (19). In Blimp-1-deficient B cells, *Xbp-1* fails to be up-regulated, resulting in impaired plasma cell function and defective antibody secretion (20). Newly generated plasma cells exit secondary lymphoid organs via *S1P1*, up-regulated by *Klf2*, and enter circulation (21, 22). Afterwards, they migrate to bone marrow under the guidance of chemokines such as C-X-C motif chemokine ligand 12 (CXCL12) (23).

While most plasma cells are short-lived, residing primarily in the medullary cords of lymph nodes and the red pulp of the spleen, a small subset migrates to the bone marrow, where they further differentiate into long-lived mature plasma cells under the influence of bone marrow-derived survival signals. Recent mechanistic advances have further elucidated the developmental continuum from germinal center B cells to LLPCs (24). Manakkat Vijay et al. identified a TIGIT^+^ transitional plasma cell precursor population generated during the late phase of the germinal center response, which preferentially gives rise to long-lived bone marrow plasma cells (24). Liu et al. demonstrated that LLPCs exhibit isotype-specific phenotypes, with IgA LLPCs being Ly6A^hi^TIGIT⁻ and IgG/IgM LLPCs EpCAM^hi^CXCR3⁻, underscoring that TIGIT expression and survival pathways are differentially regulated across isotypes (25). Collectively, these studies provide mechanistic insights into plasma cell differentiation.

### 2.2 Functions

A hallmark characteristic of plasma cells is their remarkable capacity for antibody synthesis and secretion. Based on their lifespan, plasma cells are generally classified into two main types: short-lived plasma cells, which are proliferative and survive for days to months, and LLPCs, which are non-proliferative and can persist for decades (26). LLPCs, predominantly generated throughout germinal center reactions, reside long-term in the bone marrow, where they secrete high-affinity, class-switched antibodies such as immunoglobulin G (IgG), immunoglobulin A (IgA) and immunoglobulin E (IgE) and establish stable immunological memory, enabling rapid and high antibody responses upon re-exposure to the same antigen. In contrast, short-lived plasma cells are typically formed in the extrafollicular regions of secondary lymphoid organs and predominantly generate low-affinity IgM antibodies, ultimately facilitating rapid primary immune responses (27). Other than their well-known function of antibody secretion, plasma cells are key contributors to the intricate process of immune response modulation (28). Recent studies suggest that plasma cells can inhibit the development of follicular helper T cells (28). Moreover, certain subsets of plasma cells produce anti-inflammatory cytokines such as interleukin-10 (IL-10) and IL-35, contributing to immune regulation throughout infection (29). In patients with inflammatory bowel disease, some mucosal plasma cells secrete granzyme B, exerting cytotoxic effects (30). Furthermore, IgA^+^ mucosal plasma cells have been demonstrated to facilitate tumor necrosis factor-alpha (TNF-α) and inducible nitric oxide synthase (iNOS), contributing to local inflammatory responses (31).

### 2.3 Survival and proliferation

The long-term survival of plasma cells is dependent on a specialized microenvironment, often referred to as the “niche”, which is composed of stromal and hematopoietic-derived cells (32, 33). Nevertheless, the precise cellular composition of this niche remains controversial. Multiple studies have validated that CXCL12-producing stromal cells, megakaryocytes, eosinophils, basophils, and T cells support plasma cell survival through the secretion of soluble factors (34–38). A proliferation-inducing ligand (APRIL) and IL-6 are critical pro-survival cytokines for plasma cells. Both megakaryocytes and eosinophils in the bone marrow can produce APRIL and IL-6 (35, 36), while basophils contribute to plasma cell survival *in vivo* and *in vitro* via IL-4 and IL-6 secretion (37). In addition, recent findings indicate that antibody-secreting cells (ASCs) in patients with SLE can also produce APRIL in an autocrine manner, which may further support their survival (39). Regulatory T cells (Tregs) in the bone marrow also play a critical role by expressing high levels of effector molecules. Nonetheless, deletion of CTLA-4 brings about abnormal plasma cell expansion (38). As demonstrated by Cassese et al., a combination of signaling molecules, comprising IL-5, IL-6, stromal cell-derived factor-1α (SDF-1α), TNF-α, and CD44 ligands, is essential for maintaining plasma cell longevity (40). Intrinsically, cytokines such as IL-7 and stem cell factors (SCFs) are critical in the early stages of B cell development, but fail to play a role in sustaining the survival of plasma cells. This observation suggests that plasma cells have a unique set of survival prerequisites, distinct from those of early B cells. In comparison with resting B cells, plasma cells demonstrate high expression of B cell maturation antigen (BCMA), while the expression of transmembrane activator and cyclophilin ligand interactor (TACI) and B cell-activating factor receptor (BAFF-R) is reduced, suggesting that BCMA plays a critical role in plasma cell survival (41). Although BCMA has been proposed to play a critical role in plasma cell survival by mediating APRIL- and BAFF-dependent signals (42), recent evidence suggests that BCMA is dispensable for the survival of LLPCs in mice (43). Menzel et al. demonstrated that BCMA-deficient mice maintain comparable numbers of antigen-specific LLPCs, indicating that BCMA may function primarily as a soluble decoy receptor regulating plasma cell population size rather than serving as an essential survival factor (43). Notably, while BCMA is dispensable for the survival of LLPCs, it nonetheless represents a critical therapeutic target in MM.

In addition to cytokine signaling, nutrient uptake is pivotal for sustaining the high metabolic demands of antibody production. LLPCs exhibit more enhanced glucose uptake than their short-lived plasma cells, underlining the critical role of metabolic support in maintaining their long-term survival (44). Notably, plasma cells can survive for decades in the hypoxic environment of the bone marrow. *In vitro* studies further reveal that hypoxic conditions enhance plasma cell viability, implicating hypoxia as a potential pivotal factor in promoting long-term survival of LLPCs (45).

In line with the model of replicative self-renewal, LLPCs reside in a quiescent state within the bone marrow niche. While they express a diverse array of cell cycle regulators, they can occasionally be triggered by cellular or immune signals to undergo rare and transient divisions (occurrence <1%) (46). For any given antigen-specific plasma cell, this replicative self-renewal occurs very rarely, with minimal impact on overall antibody titers (46). Nevertheless, the threshold for cell cycle re-entry is substantially reduced in cases where plasma cell precursors harbor oncogenic mutations (46). The finely regulated self-renewal mechanism becomes disrupted, with malignantly transformed plasma cells acquiring uncontrolled proliferative capacity in plasma cell disorders such as MM. This phenomenon leads to clonal plasma cell expansion in the bone marrow microenvironment, followed by further immune evasion through accumulated acquired mutations and microenvironment remodeling, ultimately entering peripheral blood.

### 2.4 Maturation and maintenance

Plasma cell maturation begins with the differentiation of activated B cells into ASCs, which transiently circulate in the blood as plasmablasts before either undergoing apoptosis or migrating to specialized tissue niches, most notably the bone marrow, where they mature into LLPCs (47). This maturation process involves morphological, transcriptional, and epigenetic changes within the bone marrow microniche (48). Joyner et al. demonstrated that early-minted blood ASCs acquire LLPC-like features through expansion of the endoplasmic reticulum, increased mitochondrial content, and upregulation of pro-survival genes (*BCL2*, *MCL1*, *BCL-XL*), conferring resistance to apoptosis (48). Duan et al. further revealed distinct LLPC maturation trajectories through single-cell transcriptomic analyses, highlighting metabolic reprogramming and the involvement of TNF–NF-κB and mammalian target of rapamycin (mTOR) signaling pathways in LLPC maintenance (47). Functionally, antibody secretion capacity increases as ASCs mature, with Nguyen et al. showing that bone marrow LLPCs produce significantly more IgG per cell than circulating early-minted ASCs (49). Importantly, Schulz et al. identified BCMA as a critical determinant of terminal plasma cell maturation (50). Using a BCMA reporter mouse, they demonstrated that BCMA expression increases with plasma cell maturity and varies by immunoglobulin isotype, emphasizing the essential role of the *in vivo* microenvironment and APRIL-mediated BCMA signaling in sustaining LLPC survival (50). Collectively, these studies underscore that plasma cell maturation and maintenance are tightly orchestrated processes integrating transcriptional, metabolic, and microenvironmental programs, ultimately ensuring the persistence of protective humoral immunity.

### 2.5 Roles in immune responses

#### 2.5.1 Roles throughout infection

Throughout the early stages of infection, plasma cells transiently emerge in peripheral blood, bolstering initial immune defense through rapid antibody secretion. Afterwards, they migrate to the bone marrow or spleen, where they differentiate into LLPCs that sustain antibody levels and confer long-term immune protection. Early studies demonstrated that infection-induced inflammatory signals cause the mobilization of plasma cells from the marrow, and this efflux reduces the size of the existing LLPC populations, with concomitant reduction in circulating antibodies derived from these plasma cell populations. This was associated with a dramatic drop in CXCL12 levels and loss of eosinophils in the bone marrow of infected mice (51). Plasma cells and their secreted antibodies play a central role in the long-term protection against chronic viral infection. During chronic or persistent infections, plasma cells undergo clonal expansion and somatic hypermutation, resulting in a diverse antibody repertoire with varying affinities and specificities, including cross-reactivity to multiple antigens (52, 53). Chronic infection is characterized by a longer-lasting germinal center reaction and a continuous differentiation of plasma cells, resulting in the emergence of higher-affinity plasma cells exhibiting increased antibody secretion rates (54). A recent report in *Science* highlighted the pivotal role of plasma cells in antibody affinity maturation, whereby clonal expansion enhances antibody affinity. This finding offers new insights for vaccine development, suggesting that the design of antigens capable of efficiently driving plasma cell expansion could represent an important strategy to strengthen immune responses (55).

As already suggested by recent investigation, the functions of plasma cells extend beyond antibody production. Specifically, certain subsets can secrete anti-inflammatory cytokines such as IL-10 and IL-35, thereby modulating immune responses throughout infection. For instance, Shen et al. reported that, a subset of B cells suppresses antimicrobial immune responses via IL-10 and IL-35 production during *Salmonella* infection (29). Phenotypic analysis identified these cells as IgM^+^CD138^hi^TACI^+^CXCR4^+^CD1d^int^Tim1^int^ plasma cells. Aside from their protective roles, plasma cells may also act as viral targets that expedite infection. Alomari et al. reported that during chronic viral infection, the differentiation of new plasma cells is involved in the early stages of viral infection in B cells, mediated by IL-21 signaling and promoting viral dissemination at early stages (56).

#### 2.5.2 Roles in vaccination and maintaining immune memory

The role of plasma cells in the immune response to vaccination is to serve as the primary source of antigen-specific antibody production, thereby mediating both the immediate and long-term humoral protection elicited by vaccines. The primary goal of vaccination is to induce the generation of plasma cells, particularly LLPCs, to provide durable humoral immunity. During natural infections, such as influenza, measles, or mumps, LLPCs can be established in the bone marrow, sustaining antibody production for decades (57). However, recent studies on SARS-CoV-2 mRNA vaccination suggest that antigen-specific plasma cells may not consistently form long-lived phenotypes in the bone marrow (57). Nguyen et al. reported that, within 2.5 to 33 months after SARS-CoV-2 mRNA vaccination, influenza- and tetanus-specific ASCs were widely detected in the LLPC compartment, whereas SARS-CoV-2-specific ASCs were primarily found in non-LLPC subsets (57). This suggests that SARS-CoV-2-specific plasma cells may not efficiently establish durable bone marrow residency. These findings indicate that the generation and maintenance of plasma cells following vaccination may differ from those induced by natural infection. Understanding the dynamics of plasma cell development and maintenance after vaccination is therefore crucial for optimizing vaccine design and improving the durability of protective immunity.

Long-lasting humoral immunity rests significantly with tightly regulated mechanisms that govern the generation, survival, and homeostasis of plasma cells. Because of their extended lifespan, LLPCs resident in the bone marrow sustain humoral immunity autonomously, independent of memory B cells, T cell help, or persistent antigen presentation. LLPCs are generated following robust germinal center reactions, which provide high-affinity, class-switched antibodies crucial for durable humoral immunity. The mechanisms by which plasma cells maintain long-term immune memory rely on specialized survival niches and extrinsic factors, as detailed in Section 2.3.

## 3 CPC formation and detection

### 3.1 Distinct immunophenotypic features of CPCs

**Figure 2:**
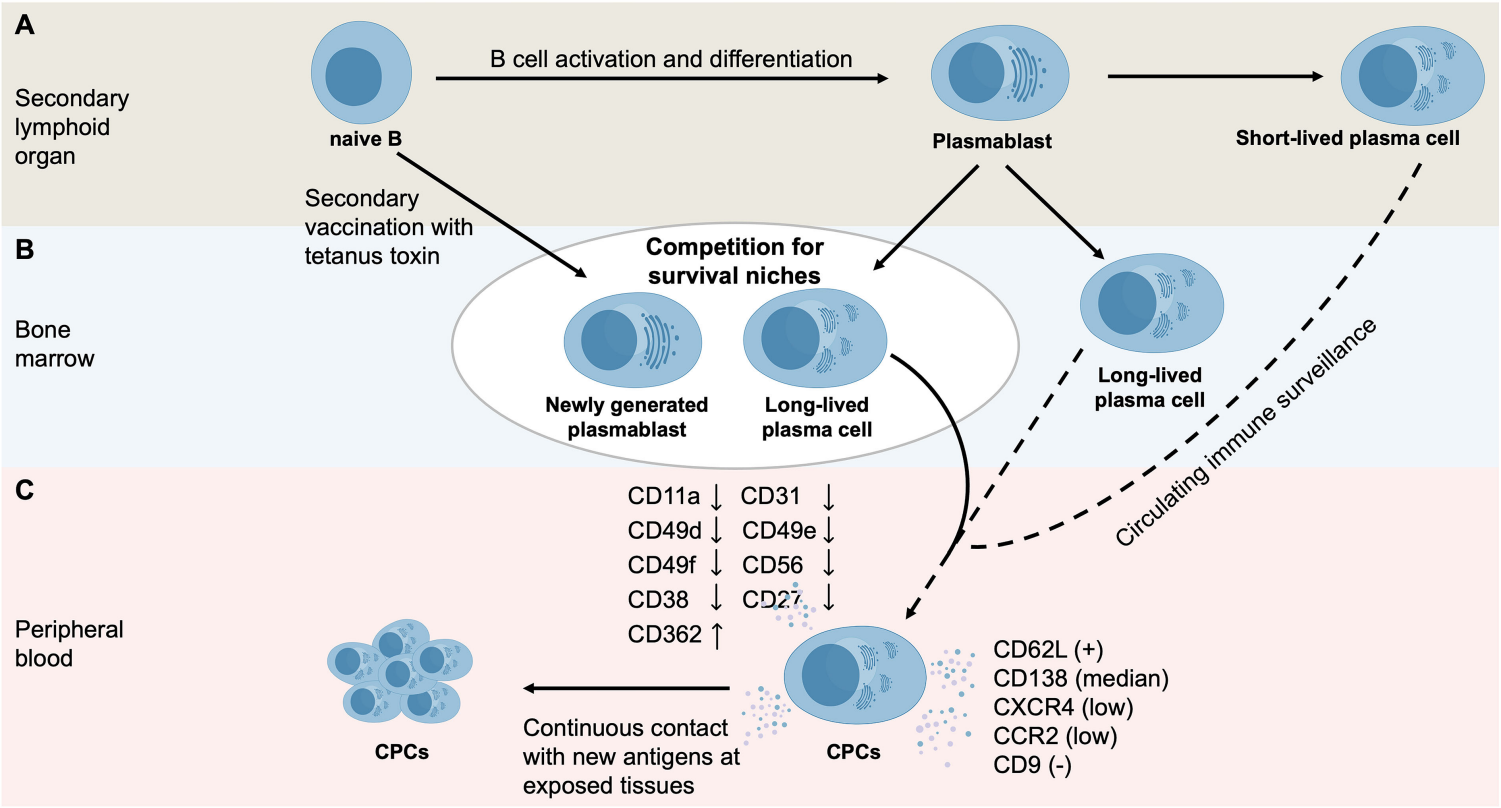
The mechanisms underlying normal CPCs formation. **(A)** In secondary lymphoid organs, antigen exposure results in B cell activation and differentiation, resulting in the generation of plasmablasts and plasma cells. Short-lived plasma cells mainly exist in peripheral lymphoid tissues, while LLPCs migrate to the bone marrow for long-term survival. Plasma cells may be released into the peripheral blood to participate in circulating immune surveillance. **(B)** Following immunization (e.g., secondary vaccination with tetanus toxin), recently generated plasmablasts must compete with resident plasma cells for survival niches in the bone marrow, driving the re-entry of resident plasma cells into the peripheral blood. **(C)** In comparison with bone marrow plasma cells, circulating normal plasma cells display a distinct immunophenotype, including: (i) reduced molecules markers, with e.g., less substantial CD11a, CD31, CD49d, CD49e, CD49f and CD56 expression; (ii) activation markers, with e.g., lower CD38 and CD27 expression; (iii) higher CD362 expression; and (iv) positive CD62L expression, intermediate CD138 levels, low CXCR4 and CCR2 levels, and absence of CD9.

Under physiological conditions, recently released CPCs progressively acquire typical plasma cell characteristics (**Figure 2**). In comparison with bone marrow plasma cells, CPCs display lower surface expression levels of adhesion molecules (CD11a, CD31, CD49d, CD49e, CD49f, and CD56) and activation markers (CD38 and CD27), while expressing higher levels of CD362 (58). In addition, CPCs differ from bone marrow plasma cells by expressing CD62L and intermediate levels of CD138, revealing lower expression of CXC chemokine receptor 4 (CXCR4) and CC chemokine receptor 2 (CCR2), and lacking CD9 expression (59). Compared with plasmablasts, CPCs display distinct phenotypic alterations, including changes in activation markers (decreased CD53, CD45, and CD9, with increased CD27), adhesion molecules (reduced CD47, CD11a, and CD50, with increased CD31, CD49d and CD329), B-cell receptor signaling molecules (decreased CD22, CD19, and HLA-DR), complement receptors (reduced CD58), co-stimulatory molecules (decreased CD40 and CD130, but increased CD86, CD272, CD126, CD32, and CD85j), and plasma cell survival–associated molecules (decreased CD268, with elevated CD270 and CD95) (58). CPCs with this immunophenotype are newly generated plasma cells migrating from lymphoid organs to the bone marrow or tissues (59).

### 3.2 Exposure to novel antigens accelerates CPC generation

Normal CPCs are undetectable in fetal umbilical cord blood (58). Nonetheless, after birth, as neonates are exposed to novel antigens in mucosal barrier tissues, such as the respiratory and gastrointestinal tracts, the number of CPCs in peripheral blood increases rapidly throughout the first weeks to months of life, reaching a peak between one and two years of age; thereafter, their numbers gradually decline throughout adulthood (58). As already demonstrated by associated studies, CPCs generally present an exceedingly activated phenotype, with 66.8~76.2% of these expressing the proliferation marker Ki-67, illustrating that local antigenic stimulation may trigger CPCs re-entry into the peripheral blood (1).

### 3.3 Competition for bone marrow niches promotes CPC re-entry into the peripheral blood

Odendahl et al. investigated the dynamics of plasmablasts and plasma cells following a secondary vaccination with tetanus toxin (2). On days 6 and 7 post-immunization, a large number of tetanus toxin-specific plasmablasts (CD19^+^/CD27^high^/intracellular IgG^high^/HLA-DR^high^/CD38^high^/CD20^–^/CD95^+^) were released into the peripheral blood from secondary lymphoid organs (2). These cells responded to chemotactic signals mediated by CXCR3 and CXCR4 ligands, probably guiding them to the bone marrow or inflamed tissue (2). Simultaneously, a population of plasma cells appeared in peripheral blood, marked by a long-lived phenotype (CD19^+^/CD27^high^/intracellular IgG^high^/HLA-DR^low^/CD38^+^/CD20^–^/CD95^+^), secreting unknown, non-tetanus toxin-specific antibodies (2). The detection of these cells in peripheral blood demonstrates that nascent plasmablasts compete with resident plasma cells for survival niches in the bone marrow (2). This competition may facilitate the re-emergence of resident plasma cells into the peripheral circulation, representing a potential mechanism contributing to CPC formation (**Figure 2**).

### 3.4 Factors influencing CPC numbers

CPC numbers are influenced by the combined effects of intrinsic cellular determinants, microenvironmental cues, and the host’s physiological or pathological state. In healthy adults, CPCs are very rare, typically exhibiting a concentration of approximately 2 CPCs per microliter of blood (1). Nonetheless, the number of CPCs can increase markedly under certain pathophysiological circumstances. Such elevations may involve either reactive plasma cells, as seen in acute responses following vaccination (2), reactive plasmacytosis triggered by viral infections (3), and autoimmune diseases such as SLE (4), or clonal plasma cells, as observed in malignant plasma cell disorders, including MM and plasma cell leukemia (5, 6). Under such circumstances, abnormal fluctuations in CPC numbers may have significant diagnostic and prognostic value for related disorders. In the context of reactive plasma cells, antigenic stimulation stands as a pivotal driver of plasma cell production, initiating and sustaining immune responses (58). The intensity and duration of these immune responses exert a direct influence on the levels of reactive CPC.

### 3.5 A possible hematopoietic stem cell-like recirculation mechanism

Hematopoietic stem cells (HSCs) are validated to circulate in the bloodstream under steady-state conditions. Circulating HSCs and their progenitors fluctuate in antiphase with the expression of the chemokine CXCL12 in the bone marrow microenvironment and follow circadian rhythms (60). These circulating HSCs have important implications for immunosurveillance (61). The bone marrow harbors specialized microenvironments for HSCs and their progenitor cells, and plasma cells, with plasma cells and HSCs sharing a similar stromal microenvironment (62). In particular, the concentration of CD34^+^ cells in the peripheral blood of healthy adults is comparable to that of CPCs under steady-state conditions (1, 63), which suggests that bone marrow plasma cells and HSCs may be regulated by similar recirculation mechanisms.

### 3.6 Detection of CPCs

#### 3.6.1 Current CPC detection methods

CPC detection techniques vary widely in sensitivity, specificity, applicability, and feasibility. Traditional cytology, immunocytochemistry (IMC), multiparameter flow cytometry (MFC), next-generation flow cytometry (NGF), allele-specific oligonucleotide quantitative PCR (ASO-qPCR), next-generation sequencing (NGS), surface-enhanced Raman spectroscopy (SERS), and inductively coupled plasma mass spectrometry (ICP-MS) each have unique strengths and limitations (**Table 1**).

**Table 1:** Advantages and disadvantages of the most frequently used methods for detection of CPCs.

Parameter	Cytology	IMF	MFC	NGF	ASO-qPCR	NGS	SERS (64, 65)	ICP-MS (66–69)
Availability	High	Low	High	High	Intermediate	Limited	High	High
Applicability	≈100%	≈100%	≈100%	≈100%	42%-75%	80%-90%	90%-95%	80%-95%
Sensitivity	<10^-2^	<10^-4^	≤10^-4^	≤2×10^-6^	≤10^-5^~10^-6^	≤1×10^-6^	≤1×10^-6^	≤1×10^-6^
Specificity	Limited	Limited	High	High	High	High	High	High
Standardized	Yes	No	Ongoing	Yes	Yes	Ongoing	Ongoing	Ongoing
Quantitative	Yes (high counts)	Yes	Yes	Yes	Yes	Yes	Yes	Yes
Sample type	Blood	Blood	Blood	Blood	Tissue	Tissue	Blood	Blood
Fresh sample	Yes	Yes	Yes (<36h)	Yes (<36h)	No	No	Yes	Yes
Sample pre-treatment	No	Yes	No	No	Yes	Yes	Yes	Yes
Time to results	<2 h	4h	2-3h	3-4h	3–4 weeks	≥7 days	4h	3-4h
Data analysis/interpretation	Subjective	Slightly subjective	Slightly subjective	Objective	Slightly subjective	Objective	Objective	Objective
CPC detection principle	DFN	Ig light-chain restriction	DFN and LAIP clonality verification	DFN and LAIP clonality verification	Patient-specific *IGH-V(D)J* gene rearrangements	Patient-specific *IGH-V(D)J* gene rearrangements	Label target proteins on the cell surface	Integrate microfluidic separation with ICP-MS-based elemental labeling detection
Relative Cost	Low	High	Intermediate	Intermediate	Intermediate	High	Low	Intermediate

This table is adapted from the study by Sanoja-Flores et al. (58). ASO-qPCR, allele-specific oligonucleotide quantitative real-time polymerase chain reaction; CTPC, circulating tumor plasma cells; DFN, different from normal; FACS, fluorescence activated cell sorting; ICP-MS: inductively coupled plasma-mass spectrometry; Ig, immunoglobulin; IGH, Ig heavy chain; IMF, immuno-fluorescence microscopy; LAIP, leukemia connected immunophenotype; MGUS, monoclonal gammopathy of undetermined significance; MFC, multiparameter flow cytometry; MM, multiple myeloma; MNC, mononuclear cells; NGF, next generation flow; NGS, next generation sequencing; NT, not tested; SERS: surface-enhanced Raman scattering; SMM, smoldering MM

Cytology remains the most accessible and inexpensive approach, but its low sensitivity (<10⁻^2^, particularly at low CPC levels), limited specificity, and inter-observer variability and the difficulty of identifying atypical CPC morphologies further restrict its reliability (70, 71). Immunocytochemical methodologies allow for more detailed characterization of monoclonal plasma cells in the blood, particularly through assessment of restricted light chain expression (72). However, it is labor-intensive and lacks standardized protocols. Flow cytometry has emerged as the primary technique for CPC detection due to its high throughput, sensitivity, reliability, and accuracy. CPCs are commonly assessed using flow cytometry in numerous MM treatment guidelines and clinical studies (73–75).

#### 3.6.2 Emerging CPC detection technologies

A wide spectrum of advanced techniques has been developed to improve both the sensitivity and specificity of CPC detection, including MFC, NGF, qPCR and NGS (58). In contrast to bone marrow aspiration cytology and core biopsy, liquid biopsy approaches, such as the analysis of CTCs and circulating tumor DNA (ctDNA), are increasingly used in hematologic malignancies due to their minimally invasive nature and compatibility with multiple detection platforms. Of these methods, MFC plays a central role in differential diagnosis by providing relatively rapid and reliable results, and is used to distinguish malignant from reactive conditions and the classification of multiple diseases such as MM and MGUS (76–78). The standardization of flow cytometry for CPC detection has improved significantly in recent years. Collaborative initiatives, such as the EuroFlow consortium, have established standardized antibody panels, sample preparation protocols, and quality control guidelines, greatly reducing inter-laboratory variability. However, it requires fresh samples within 36 hours and specialized instrumentation. NGF further enhances sensitivity (≤2×10^-6^) and has benefited from international standardization efforts such as EuroFlow. In contrast to conventional flow cytometry and immunocytochemistry, NGF approximately doubles the detection rate of CPCs in peripheral blood (MGUS: 19%-37% vs. 59%; SMM: 15%-50% vs. 100%; MM: 50%-73% vs. 100%) (79). However, it should be noted that the 100% detection rates reported for SMM and MM are based on specific studies and may not be universally applicable. Other studies have reported lower detection rates for CPCs in these conditions, highlighting variability across NGF-based assessments. In terms of prognosis, CPC quantification by NGF has been useful for effectively distinguishing high-risk MGUS cases that are likely to progress to MM from low-risk cases, and in anticipating survival outcomes in NDMM patients. However, in a subset of MM patients, morphology assessment alone is insufficient to detect CPCs among 2,000 analyzed cells per smear, especially when the level of peripheral blood infiltration is below 0.1%. At the same time, the implementation of NGF demands substantial technical expertise and costly infrastructure. Molecular approaches, including ASO-qPCR and NGS, provide high specificity and detailed molecular resolution, yet are limited by complex sample preparation, restricted availability, longer turnaround times, and higher costs.

As artificial intelligence (AI)-assisted digital pathology continues to evolve, AI-driven automated cellular analysis has emerged as a rapidly advancing field. Chinese researchers have developed the AI-based Morphogo system for digitizing peripheral blood smear samples (70). This system identifies and classifies nucleated cells (approximately 500 to 2000 cells per smear), demonstrating superior sensitivity (89.03%), specificity (99.68%), and accuracy (99.64%) in CPC detection, and facilitates efficient CPC screening in MM patients. Integration of AI with surface-reinforced Raman spectroscopy (SERS) further enhances CPC detection sensitivity. SERS employs gold-coated magnetic nanoparticles functionalized with anti-CD138 and anti-CD38 antibodies to detect CPCs in peripheral blood. Machine learning algorithms applied to SERS signals have proven effective in identifying MM patients with high accuracy (64). Furthermore, the inductively coupled plasma mass spectrometry (ICP-MS), as a high-throughput analytical technique that integrates inductively coupled plasma with mass spectrometry, can overcome major limitations in the detection of CPCs, such as the rarity, heterogeneity, and interference from complex blood matrices (e.g., leukocytes) (66), when combined with microfluidic chip technology. This integrated approach enables the high-purity isolation of CPCs and significantly improves the detection rate of CPCs in clinical samples (100%) (67–69). It offers rapid analysis (5 minutes per 1 mL of blood) and cost efficiency (66) (**Table 1**).

Beyond AI-assisted cytology, sequencing-based approaches provide complementary insights into CPC biology and tumor burden. Sequencing workflow to interrogate few tumor cells (SWIFT-seq), a single-cell sequencing workflow, applying single-cell RNA and B cell receptor sequencing to paired bone marrow and CTC samples from MM patients, this approach allows detection of cytogenetic abnormalities, assessment of proliferative indices, and tracking of clonal dynamics (80). A circulatory dynamics model incorporating tumor burden, proliferation, cytogenetics, and circulatory capacity can further explain CTC levels in blood. **Table 1** clearly summarizes the relative advantages and limitations of these emerging methods, highlighting key factors such as cost, time to results, sample type, and standardization status.

## 4 CPCs and plasma cell disorders

### 4.1 Pathogenesis of plasma cell disorders

Plasma cell disorders are a group of clonal plasma cell proliferative diseases, comprising monoclonal gammopathy of undetermined significance (MGUS), MM, plasmacytomas (solitary bone plasmacytoma and extramedullary plasmacytoma), immunoglobulin deposition diseases (primary light chain amyloidosis, light and heavy chain deposition disease), and POEMS syndrome (73). The central feature of plasma cell disorders is the clonal expansion of premalignant or malignant plasma cells, characterized by monoclonal immunoglobulin secretion (81, 82). The tumor microenvironment (TME) plays a critical role in the initiation and progression of plasma cell disorders. The TME is a highly dynamic and complex microenvironment that facilitates tumor growth, increases drug resistance, and compromises immune surveillance (83). Immune remodeling within the bone marrow microenvironment significantly accelerates the progression of plasma cell disorders. Various cellular components function redundantly and compensatory to support the survival of malignant plasma cells. Bone marrow mesenchymal stem cells (BM-MSCs) are critical participants, MSCs secrete CXCL12, the ligand for CXCR4, facilitating plasma cell homing to the bone marrow, providing contact-dependent support through integrins, and secreting pro-survival, anti-apoptotic, and pro-angiogenic cytokines such as IL-6, vascular endothelial growth factor (VEGF), and insulin-like growth factor 1 (IGF-1). In addition to MSCs, other factors promoting disease progression comprise the progressive impairment of tumor-suppressive immune cells (e.g., anti-MM T cells) and the accumulation of pro-tumorigenic immune cells, such as Tregs, Th17 cells, tumor-associated macrophages (TAMs), myeloid-derived suppressor cells (MDSCs), and immunosuppressive dendritic cells (DCs) (84). Plasma cell disorders are also characterized by extensive chromosomal abnormalities, which are commonly present from the early stages of the disease. For instance, structural centrosome aberrations may drive early aneuploidy and contribute to malignant transformation (81). Regarding clonal evolution, the transformation of post-germinal center B cells or plasma cells into MGUS and subsequently into MM involves an initiating event followed by multiple secondary genetic alterations. Initiating events typically comprise immunoglobulin heavy chain (IgH) translocations or hyperdiploidy, while secondary events include copy number variations, somatic mutations, and epigenetic alterations, all of which are advantageous for disease progression. Therefore, the clonal evolution from MGUS to MM is driven by a combination of secondary translocations, copy number alterations, oncogenic mutations, epigenetic changes, and tumor microenvironmental remodeling in premalignant plasma cell clones (85).

### 4.2 The generation of CPCs in plasma cell disorders

**Figure 3:**
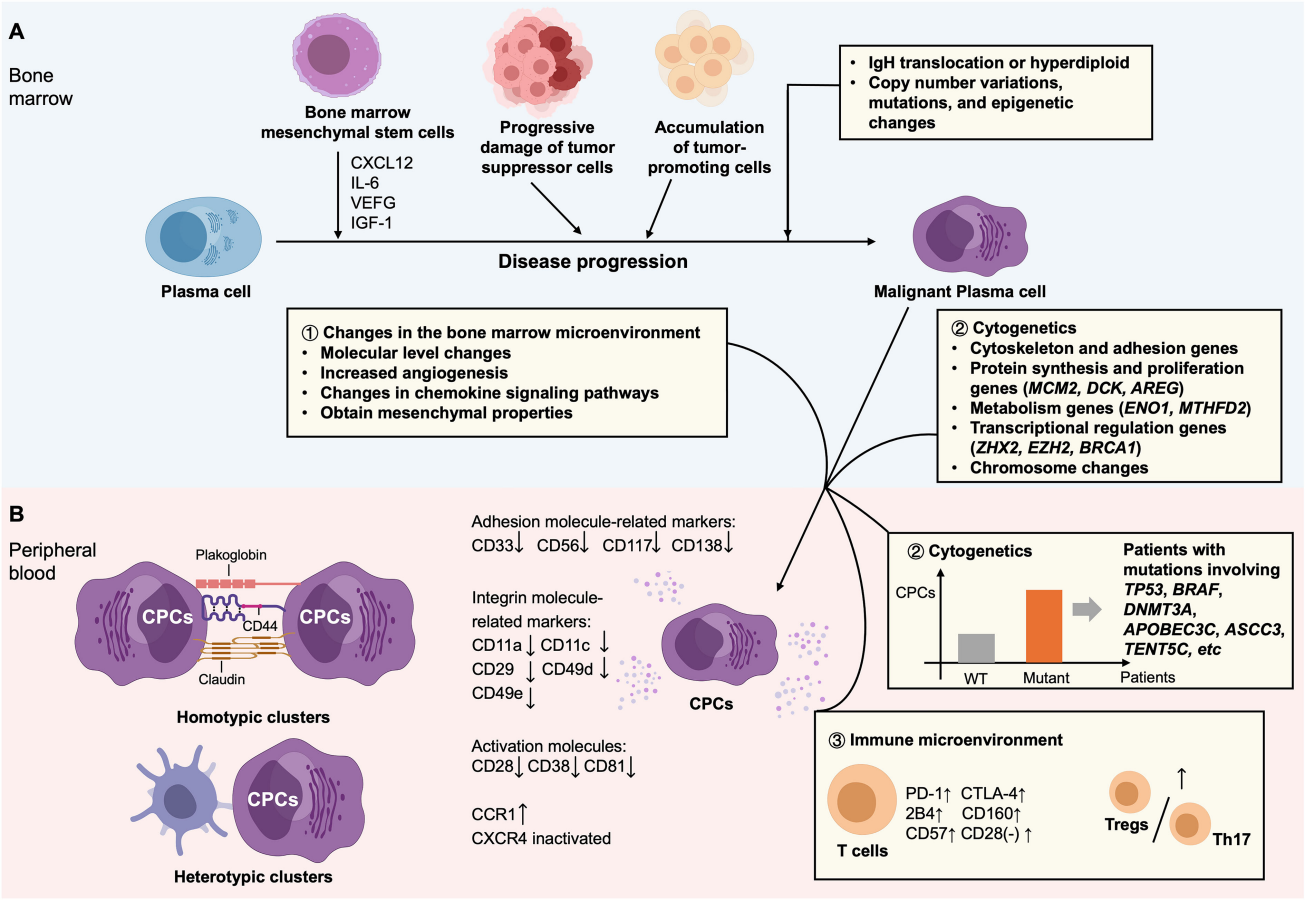
Proposed mechanisms driving the formation of CPCs in tumor. **(A)** Multiple factors within the bone marrow microenvironment that facilitate the malignant transformation of plasma cells: (i) a complex cellular milieu supports the expansion and survival of malignant plasma cells, with bone marrow-derived mesenchymal stem cells playing a key role; (ii) progressive impairment of tumor-suppressive cell populations, accompanied by the enrichment of tumor-promoting cells subsets; and (iii) multiple genetic events, initiating with primary events such as immunoglobulin heavy chain (IgH) translocations or hyperdiploidy, and followed by secondary events comprising copy number variations, somatic mutations, and epigenetic modifications. **(B)** Mechanisms facilitating the dissemination of malignant plasma cells from the bone marrow into the peripheral circulation: (i) remodeling of the tumor microenvironment, including alterations at the molecular level, increased angiogenesis, dysregulation of chemokine signaling pathways, and acquisition of mesenchymal-like properties; (ii) cytogenetic aberrations, involving the involvement of hub genes that may enable malignant plasma cells to survive and proliferate outside of the bone marrow microenvironment, as well as specific gene mutations and chromosomal alterations; (iii) immune microenvironmental dysregulation, particularly impaired T-cell function, characterized by T-cell exhaustion or senescence, and an imbalance between Tregs and Th17 cells; and (iv) dissemination mechanisms resembling those of CTC clusters, promoting metastasis formation, which are implicated in the initiation and promotion of distant metastases.

Throughout the progression of plasma cell disorders, malignant plasma cells migrate from the bone marrow into the peripheral blood, forming CPCs, which play a key role in tumor dissemination (73). Multiple studies have investigated the mechanisms of CPC generation in MM. The generation of CPCs in MM patients may involve three primary mechanisms (**Figure 3**).

#### 4.2.1 Tumor microenvironment

Alterations in the bone marrow microenvironment promote the egress of malignant plasma cells into the peripheral circulation. Current models of MM dissemination suggest that regions of severe hypoxia and pro-inflammatory conditions within, the bone marrow drive malignant plasma cells into a quiescent state, prompting their migration into the circulation to access alternative supportive niches (86). In comparison with intramedullary myeloma cells, CPCs exhibit decreased expression of adhesion molecules, integrins and activation molecules related markers. These alterations, along with elevated angiogenesis, disrupt adhesion between malignant plasma cells and the bone marrow endothelium, facilitating their intravasation into peripheral blood (87, 88). Chemokine signaling alterations are also critical in extramedullary dissemination. More importantly, hypoxia-inducible factor-2α (HIF-2α) is activated during chronic hypoxia, inducing expression of chemokine receptor CCR1 in MM cells. The interaction between up-regulated CCR1 and inactivated CXCR4 signaling (the CCR1/CXCR4 axis) promotes the egress of MM cells from the marrow, thereby facilitating to dissemination (89). Additionally, epithelial-derived CPCs undergo epithelial to mesenchymal transition-mesenchymal-to-epithelial transition (EMT-MET), characterized by changes in cell adhesion, motility, invasiveness, and loss of epithelial markers, transforming into mesenchymal-like cells that acquire mesenchymal traits enabling vascular invasion (90).

#### 4.2.2 Cytogenetic alterations

CPCs share similar genetic profiles with bone marrow plasma cells. MinimuMM-seq analysis of enriched CPCs demonstrated that CPCs are malignant, and exhibit copy number abnormalities consistent with bone marrow samples (91). Another study employing second-generation flow cytometry analyzed 116 matched samples (55 bone marrow, 53 peripheral blood, and 8 extramedullary plasmacytomas), and demonstrated that approximately 22% of CPCs may originate from distant marrow sites (92). Moreover, 86% and 87% of mutations detected in bone marrow and plasmacytoma cells, separately, were also present in CPCs. 82% of bone marrow mutations were also detected in CPCs, as evidenced by Gene expression analysis of paired CPCs and bone marrow plasma cells in individual patients. Cytogenetic abnormalities may confer plasma cells with immune evasion capabilities and enhanced proliferative potential, thereby facilitating their entry into circulation. Recent studies have identified 41 hub genes involved in CPC biology (93). In addition to genes associated with cytoskeleton and adhesion, these comprise genes involved in protein synthesis and proliferation (e.g., *MCM2*, *DCK*, *AREG*), metabolic processes such as glycolysis and lactate production (e.g., *ENO1*, *MTHFD2*), and transcriptional regulation (e.g., *ZHX2*, *EZH2*, *BRCA1*), many of which may promote plasma cells to survive independently of the bone marrow microenvironment (93). A study employing next-generation sequencing (NGS) in a Chinese cohort showed that patients harboring mutations in genes such as *TP53*, *BRAF*, *DNMT3A*, *APOBEC3C*, *ASCC3*, and *TENT5C* exhibited significantly higher CPC levels (94). Down-regulation of TP53 may lower expression of E-cadherin and simultaneously facilitate EMT regulators, eventually lessening cell adhesion to the extracellular matrix. TP53 loss may also up-regulate microRNA-19a/CXCR5 signaling, enhancing invasiveness of myeloma cells (95). Additionally, CPCs have been linked to several chromosomal abnormalities, including t(4;14), del(13q), del(17p), t(11;14), and t(14;16) (58). Among these, high-risk cytogenetics—particularly del(17p13)—are strongly associated with increased CPC counts and play a key role in the development of secondary plasma cell leukemia (PCL), when it emerges as a novel cytogenetic finding during clonal evolution (96).

#### 4.2.3 Immune microenvironment

Immune dysfunction, particularly impaired T cell activity, may facilitate their escape into peripheral blood because malignant plasma cells can be recognized and eliminated by cytotoxic T cells (84). As shown in (97), in MGUS, T cell clusters are enriched in stem-like memory (TCF1^hi^) and tissue-resident memory phenotypes. The persistence of these populations may be crucial for maintaining tumor immune surveillance throughout the MGUS stage. In contrast, MM is marked by a loss of TCF1^+^ memory T cells and elevated expression of cytolytic and senescence markers in T cells, indicating a breakdown of protective immunity and immune surveillance. Enhanced T-cell senescence and exhaustion in MM have been confirmed in other studies as well. Markers correlated with T-cell exhaustion (PD-1, CTLA-4, 2B4, CD160) and senescence (CD57, and loss of CD28 expression) are significantly up-regulated in both the peripheral blood and bone marrow of MM patients, where a more pronounced elevation was observed in bone marrow T cells (98). Therefore, the immunosuppressive tumor microenvironment contributes to immune evasion in MM (98). Additionally, T-helper 17 (Th17) cells can impair tumor immune surveillance through cytokine secretion (84). The Treg/Th17 cell ratio, a key indicator of immune regulation, is significantly increased in MM compared with MGUS, supporting the presence of a more immunosuppressive milieu in MM (99).

In addition, CPCs may contribute to metastasis formation through mechanisms like those observed in circulating tumor cell (CTC) cluster formation. These clusters can be homotypic, which consist solely of tumor cells, or heterotypic, which involve tumor cells in association with other cell types. Homotypic CTC clusters form characteristic oligoclonal aggregates, in which cell adhesion molecules such as plakoglobin, claudins, and CD44 are collectively critical for maintaining intercellular connections (100). Hypoxic conditions have been demonstrated to upregulate the expression of these molecules and accelerate cluster formation (100). These homotypic clusters can lead to epigenetic alterations, such as hypomethylation at binding sites for OCT4, NANOG, and SOX2, thereby conferring stem cell-like properties that facilitate metastasis (100). Heterotypic CTC clusters, formed between tumor cells and other cell types such as cancer-correlated fibroblasts, or platelets, display enhanced proliferative, invasive, and homing capabilities at metastatic sites (100). Furthermore, these clusters are also more resistant to immune surveillance (100). Similarly, CTCs interact extensively with a vast spectrum of immune cells. They form CTC-neutrophil and CTC-MDSCs clusters that promote extravasation, differentiation and proliferation. CTC clusters can modulate dendritic cell function and exhibit exceptional resistance to natural killer (NK) cell cytotoxicity or evade NK cell attack through specific molecular pathways. CTCs can directly interact with CD4^+^ helper T cells and CD8^+^ cytotoxic T cells, initiating immunosuppressive responses that support tumor cell survival (101). CPCs may adopt similar mechanisms, interacting with diverse immune cells to escape immune surveillance and facilitate dissemination and metastasis. Therefore, elevated CPC levels may reflect impaired immune function and an increased risk of tumor progression and metastasis.

In summary, malignant plasma cells in MM can enter the peripheral circulation by acquiring microenvironmental remodeling capabilities, genetic abnormalities, and immune escape mechanisms. Conversely, MGUS represents an earlier disease stage in which these mechanisms are not fully developed. For instance, while effector immune function is progressively impaired in both MGUS and MM (84), the immune abnormalities in MGUS are milder and may be insufficient to enable CPCs escape into peripheral blood.

### 4.3 CPCs in diagnosis, prognosis and treatment of plasma cell disorders

#### 4.3.1 Early detection

CPCs can be detected in the peripheral blood from the earliest stages of premalignant transformation, with levels varying across various stages of plasma cell disorders, including MGUS, Smoldering Multiple Myeloma (SMM), MM, and PCL. CPCs analysis offers a minimally invasive, low-risk, and reproducible approach for assessing early disease progression risk.

In MGUS, about 1% of patients progress annually to malignant disease (58), highlighting the need for early monitoring. A European study showed that a CPC level of ≥0.058 cells/μL in peripheral blood could differentiate MGUS from MM with 88% accuracy and was associated with significantly higher 30-month progression risk (79). Similarly, a prospective study of 254 asymptomatic patients in Athens demonstrated that CPCs positivity was significantly associated with increased risk of progression to symptomatic MM (HR: 2.99, P = 0.024) (102).

In SMM, elevated CPC levels in peripheral blood are both indicative of malignant transformation and predictive of disease progression in SMM (73). The multicenter iMMunocell study reported that patients with CPCs >0.015% displayed a significantly higher progression rate in contrast to those with CPCs ≤0.015% (37.5% vs. 4%, P < 0.001) (103). Retrospective data further confirmed that patients fulfilling high CPC levels criterion displayed a significantly elevated risk of progression to active MM within 2 years (71% vs. 24%, P = 0.001) and 3 years (86% vs. 34%, P < 0.001), in contrast to those with lower CPC levels (104).

In more advanced disease, primary plasma cell leukemia (pPCL) is characterized by markedly increased plasma cells in peripheral blood, and its diagnostic criteria have been revised in recent years. In 2013, the International Myeloma Working Group (IMWG) defined pPCL as CPCs ≥20% and/or ≥2×10^9^/L (105). Subsequent evidence showed that patients with ≥5% CPCs had similarly poor outcomes and higher pPCL detection rates, leading the IMWG in 2021 to lower the threshold to ≥5% (6, 106). More recently, Czech researchers used multiparameter flow cytometry and proposed a lower threshold of CPCs ≥2% to identify a subset of ultra-high-risk newly diagnosed MM (NDMM) patients with pPCL-like characteristics (107). These findings suggest that future refinements may adopt CPCs ≥2% as a diagnostic threshold to facilitate earlier detection and intervention.

#### 4.3.2 Prognosis

##### 4.3.2.1 Risk stratification

Accurate risk stratification is essential for prognosis and treatment planning. In SMM, the iMMunocell multicenter study identified 0.015% CPCs as a critical threshold for risk stratification and proposed the “20/2/0.015” SMM model (defined by serum free light chain (sFLC) ratio >20, M-protein >2 g/dL, CPCs >0.015%), which outperformed the traditional 20/2/20 system (103). In addition, CPCs should be taken into consideration alongside conventional markers such as sFLC ratio and M-protein throughout SMM diagnosis to better identify high-risk patients (73).

Similarly, in MM, CPCs have been shown to enhance existing systems. An Italian cohort showed that R-ISS II patients with ≥1 CPCs had worse outcomes than CPC-negative patients, but better than R-ISS III patients. Likewise, in a single-center study of 336 NDMM patients, adding CPCs ≥0.05% improved the discriminatory power of R2-ISS (108). A meta-analysis in China confirmed that elevated CPC levels correlate with advanced ISS/R-ISS stages and high-risk cytogenetic, indicating more aggressive disease features (5). Furthermore, a novel prognostic algorithm integrating CPCs, the Phenotypic Classification System (PCS) and R-ISS achieved superior accuracy compared with any individual system or pairwise combination in stratifying NDMM risk (109).

##### 4.3.2.2 Prognostic prediction

Beyond risk stratification, CPCs themselves are recognized as an independent adverse prognostic factor in NDMM (110). Several studies have proposed effective thresholds: CPCs ≥0.165% predicted inferior OS, 71% vs. 87% in CPCs <0.165% at 3 years (111); CPCs ≥0.105% was linked with markedly shorter PFS and OS (110); CPCs ≥0.038% effectively distinguished high tumor burden and low remission rate populations, and served as an independent predictor of PFS and OS (112). Over the past two years, numerous in-depth studies further validated the prognostic utility of CPCs in MM. As reported by a comprehensive meta-analysis, increased CPC levels were significantly correlated with shorter OS and PFS across all subgroups, irrespective of geographic region, sample size, cutoff values, detection timing, initial treatment regimens, or data types (5). A prospective multicenter study also revealed that CPC-negative NDMM patients at diagnosis had a 5-year OS of 42%, in contrast to 25% in CPC-positive patients (P < 0.05), with prognosis determined more by the mere detectability of CPCs than by their absolute levels (113). In a study of Chinese patients, those with ≥2% CTCs showed significantly worse PFS (P < 0.001; 49 months vs. 25 months) and OS (P < 0.001; NR vs. 38 months) compared to those with <2% CTCs, indicating that a 2% CTC threshold might serve as an indicator of ultra-high-risk MM (114). CPC levels in MM patients are also linked to minimal residual disease (MRD). Patients with CPCs >0.02% had a 2-year MRD-negative rate of 45%, in contrast to 67% and 74% for patients with <0.02% CPCs or undetectable CPCs, respectively (115). Moreover, the median time to achieve MRD negativity was significantly longer in the high CPC group than in the low CPC and CPC-negative groups (34 vs. 17 vs. 13 months, P < 0.001) (79). In PCL, CPCs likewise demonstrate significant prognostic value. In a study involving 33 patients suffering from primary and secondary PCL, complete loss of CD20 expression on CPCs was associated with increased mortality (116). Patients with ≤5% CD20^+^CPCs had significantly shorter OS than those with >5% CD20^+^ CPCs (3.4 vs. 47.4 months, P = 0.044). Further studies are required to validate this finding (116).

#### 4.3.3 Correlation with treatment outcome

A prospective study of 141 NDMM patients assessed CPC levels at diagnosis and after 3 (Peripheral blood MRD, PBMRD1) and 6 cycles (PBMRD2) of chemotherapy (117). PBMRD positivity, defined as CPCs ≥0.0001%, was significantly associated with inferior event-free survival and OS, whereas PBMRD negativity independently predicted favorable event-free survival at any time. These findings support routine CPCs monitoring after MM chemotherapy to identify patients at risk of poor response.

Autologous hematopoietic stem cell transplantation (auto-HSCT) remains a cornerstone in the treatment of MM. Multiple studies have demonstrated that CPC status before and after auto-HSCT serves as an independent prognostic factor for PFS, OS, and TTP (118–120). Accordingly, the Chinese expert consensus recommends CPC testing in peripheral blood before auto-HSCT and at day 100 post-transplant (73). In line with this, Chakraborty et al. stratified patients into four groups according to CPC status at diagnosis and pre-transplant: CPC−/−, CPC−/+, CPC+/−, and CPC+/+ (120). In multivariate analysis for overall survival, the CPC-positive groups exhibited substantially higher risk of death compared with the CPC−/− group. Importantly, the prognostic impact of CPCs extends to both transplant-eligible (TE) and transplant-ineligible (TI) NDMM patients. A Greek study including both TE and TI NDMM patients identified an optimal cutoff of 0.02% (115). Patients with CPCs ≥0.02% showed significantly shorter median PFS compared with CPCs <0.02%. Multivariate analysis further confirmed that elevated CPCs above this threshold had an independent prognostic impact on PFS, conferring even greater risk of progression than ISS stage III or high-risk cytogenetics.

#### 4.3.4 Recent therapeutic advance

Recent advancements in therapeutic approaches have significantly improved outcomes for MM patients, especially those with relapsed or refractory disease. Novel therapeutic strategies include monoclonal antibodies, small molecules, and autologous cell-based immunotherapies such as chimeric antigen receptor T-cell (CAR-T) therapy and bispecific antibodies (121). To date, BCMA-targeted CAR-T therapy has shown remarkable efficacy for MM treatment (122), with two FDA-approved products: Idecabtagene vicleucel (ide-cel) and Ciltacabtagene autoloeucel (cilta-cel) for relapsed/refractory MM (121). Additional therapeutic agents include CD38-targeting antibodies (daratumumab, isatuximab), BCMA-targeted agents (belantamab mafodotin, teclistamab), immunomodulatory drugs (lenalidomide, pomalidomide), proteasome inhibitors (bortezomib, carfilzomib), monoclonal antibody elotuzumab, exportin 1 (XPO1) inhibitor selinexor, and various vaccine-based therapies (121, 123, 124). Approved both domestically and internationally, these treatments now provide clinicians with more than ten therapeutic options. The clinical utility of CPCs within the context of these innovative therapies warrants further investigation. At the 2024 ASH Annual Meeting, researchers from the Netherlands presented findings from the Perseus study, identifying CPCs>0.175% as a biomarker for poor prognosis in transplant-eligible, high-risk NDMM patients, who received bortezomib, lenalidomide and dexamethasone with or without daratumumab throughout induction/consolidation, and lenalidomide with or without daratumumab during maintenance (125). In addition, PBMRD monitoring, alongside bone marrow MRD assessment, has been increasingly recognized as an important tool for evaluating novel therapies in MM, with the potential to serve as an independent indicator in the future. Nonetheless, several pivotal questions remain unaddressed despite these promising findings. For instance, while CPCs are thought to arise from malignant bone marrow cells migrating into peripheral circulation, few studies have directly investigated how bone marrow tumor burden correlates with CPC levels. Moreover, recent research has underscored the potential of circulating microRNAs (miRNAs) and cell-free DNA (cfDNA) as biomarkers for MM diagnosis and prognosis (126–128). Given that CPCs, miRNAs, and cfDNA all serve as indicators in liquid biopsy, future studies should investigate their potential interplay, which could provide new insights into MM pathophysiology and risk stratification.

#### 4.3.5 CPCs as clinical biomarkers

In MM, malignant plasma cells exhibit patchy infiltration within the bone marrow. Studies have confirmed that cytogenetic alterations and mutations vary across different tumor sites, reflecting the spatial genetic heterogeneity (129). Although bone marrow plasma cell assessment remains the gold standard for evaluating tumor burden in MM patients, biopsy from a single bone marrow site cannot fully capture the spatial genetic heterogeneity of the disease and may fail to reflect the full disease heterogeneity. Malignant plasma cells in MM disseminate from diverse bone marrow regions or extramedullary sites into the peripheral blood. As a result, peripheral blood sampling may provide a more comprehensive reflection of the overall tumor burden when compared to samples acquired from a single-site bone marrow aspiration. Genetic analyses support this view, revealing that CPCs in peripheral blood offer genetic insights comparable to those of bone marrow plasma cells and may even be undetectable in bone marrow specimens (130). Although approximately 15% of MM patients demonstrate phenotypic discordance between bone marrow clonal plasma cells and matched CPCs, studies have confirmed and extended this finding from a phenotypic standpoint. This discrepancy is more prevalent in patients with elevated CPC levels, suggesting that the increase in CPCs may result from the dissemination of myeloma cells with distinct phenotypic (and possibly genetic) features from diverse bone marrow compartments into the peripheral circulation (115). Overall, for tumor burden assessment, CPC detection in peripheral blood provides a more comprehensive assessment compared to single-site bone marrow biopsy, offering advantages such as simpler sample acquisition, minimal invasiveness, and high reproducibility.

### 4.4 International consensus and advances in MM-related CPC assessment

As clinical research advances and new evidence accumulates, recommendations regarding CPCs in peripheral blood have undergone multiple updates in both national and international clinical guidelines. In China, the 2015 edition of the MM diagnosis and treatment guidelines was the first to include the percentage of peripheral blood CPCs as a mandatory assessment parameter. This requirement has been retained in all subsequent versions (2017, 2020, 2022, and 2024), with the 2024 edition further incorporating CPCs percentage into the MM prognostic stratification system (73). Specifically, patients with CPCs ≥2% are classified as ultra-high risk, while those with CPCs ≥0.07% are categorized as high-risk (73).

Internationally, the 2023.v2 edition of the National Comprehensive Cancer Network (NCCN) guidelines recognized CPCs as a high-risk clinical factor in MM. In the updated 2025.v1 version, CPCs have also been recognized as a risk factor for MM relapse (131). IMWG included elevated peripheral blood CPCs as a potential diagnostic marker in their 2014 diagnostic criteria (132). In 2021, the IMWG published a consensus on pPCL, revising its diagnostic threshold to CPCs ≥5% (6). Furthermore, the 2022 and 2024 guidelines on MM management both identified elevated peripheral blood CPC levels as a high-risk feature for SMM (133, 134). Furthermore, a 2021 consensus statement by the European Myeloma Network also acknowledged the prognostic relevance of CPCs in peripheral blood (135).

## 5 CPCs and autoimmune diseases

### 5.1 Pathogenesis of autoimmune diseases

Autoimmune diseases are characterized by aberrant immune responses in which the immune system mistakenly targets self-tissues, leading to organ damage or dysfunction. Representative examples include SLE, rheumatoid arthritis (RA), and multiple sclerosis. Their pathogenesis involves a complex interplay of genetic predisposition, environmental triggers, and dysregulated immune mechanisms, among which B cells play a pivotal role. Disease activity in autoimmune disorders is closely associated with autoantibodies secreted by plasmablasts and plasma cells. During disease flares, waves of newly generated autoreactive plasma cells might contribute to the occupation of plasma cell niches by autoreactive LLPCs, replacing old, protective plasma cells. CPCs play a central role in the pathogenesis of autoimmune diseases by continuously producing autoantibodies, which are directly implicated in the initiation and perpetuation of chronic inflammation and tissue damage. Plasma cells, particularly the long-lived subset, can escape normal tolerance checkpoints and persist in the circulation and inflamed tissues, secreting high-affinity autoantibodies that target self-antigens. The sustained presence of these autoantibodies leads to immune complex formation, complement activation, and recruitment of inflammatory cells, thereby driving chronic inflammation and organ-specific or systemic tissue injury (136, 137). In comparison with short-lived plasma cells, LLPCs exhibit marked resistance to conventional therapies and persistently secrete pathogenic antibodies, which drive the chronicity and relapse of autoimmune diseases (138).

Clinical observations further support the pathogenic role of plasma cells in autoimmunity, as approximately 30–35% of patients with lupus and RA show elevated levels of plasma cells (139). Consequently, effective depletion of autoreactive plasma cells may represent a key strategy for curative treatment of autoimmune diseases. However, although B cell depletion therapy can eliminate most circulating B cells in peripheral blood (including CPCs), clinical outcomes vary considerably among individuals, likely due to differential activation or survival signals for B cells provided by tissue microenvironment.

### 5.2 CPCs in SLE prognosis and treatment monitoring

SLE is a chronic inflammatory autoimmune disease characterized by hyperactivation of B cells and an increased frequency of CPCs (4). Simultaneously, terminally differentiated B cells, namely plasmablasts and LLPCs, experience clonal expansion and secrete large amounts of autoantibodies. These antibodies not only mediate the formation of immune complexes but also trigger the production of pro-inflammatory cytokines through type I interferon signaling pathways. This cascade exacerbates tissue injury, sustains a protracted inflammatory milieu, and leads to multi-organ involvement in affected patients (140). Previous studies have suggested that circulating plasmablasts serve as biomarkers for assessing SLE disease activity, anticipating disease flares, and guiding therapeutic decisions (141–143). Numerous research supports the potential role of CPCs in both disease monitoring and treatment evaluation in SLE (144–146). Deng-Ho et al. reported that the percentage of peripheral CD27^high^ plasma cells significantly correlates with SLE Disease Activity Index (SLEDAI) scores, anti-dsDNA antibody titers, and complement (C3/C4) levels in non-infected SLE patients (144). Nevertheless, this association was no longer observed in those SLE patients with concurrent infections, suggesting that the frequency of peripheral CD27^high^ plasma cells may serve as a differentiation marker to distinguish between lupus flares and infections. Moreover, data from several phase III clinical trials of belimumab analyzed by Parodis et al. suggested that treatment responders exhibited a greater decrease in peripheral CD19^+^CD20⁻CD138^+^ LLPCs in comparison with non-responders (−48.2% vs. −37.1%; *P* = 0.024) (145). In a comparative analysis of patients receiving belimumab versus placebo, only the treatment group exhibited a rapid decline in LLPCs, indicating a greater protective effect against disease flares (146). Notably, this trend was absent in the placebo cohort (146). Taken together, these findings suggest that monitoring CPC levels may function as a useful tool for evaluating treatment response and anticipating disease relapse in SLE. Nonetheless, further studies are imperative to validate these observations.

## 6 Conclusions

In summary, the literature indicates that CPCs may enter the peripheral blood from the bone marrow stroma due to multiple factors, among which tumor microenvironment and cytogenetic abnormalities play crucial roles in this process. The presence of CPCs represents a valuable approach for assessing disease status and treatment efficacy. In plasma cell neoplasms, particularly MM and PCL, CPCs have been firmly established as important prognostic and diagnostic biomarkers. In MM, quantification of CPCs is now recognized as a key risk stratification tool, with thresholds as low as 0.038% predicting adverse outcomes, whereas ≥0.02% correlated with lower MRD negativity, and ≥2% CPCs as a marker of ultra-high-risk MM with pPCL-like features. Although the current diagnostic criterion for pPCL is ≥5% CPCs in peripheral blood, accumulating evidence suggests that even lower levels are clinically relevant and should be considered in the design of future clinical trials. In autoimmune diseases, the role of CPCs remains poorly defined. Although plasma cells are pivotal for autoantibody production and disease pathogenesis in conditions such as SLE, current research predominantly emphasizes tissue-resident and LLPCs rather than circulating subsets. Investigations into plasma cell heterogeneity and targeted depletion strategies are ongoing; however, the clinical utility of CPC quantification outside hematologic malignancies is still limited. Technological advances have improved sensitivity and standardization of CPC detection, enabling minimally invasive monitoring and investigation of CPC biology. Future research should prioritize the optimization and standardization of CPC detection methodologies. Furthermore, integrating these findings into precision medicine and targeted therapeutic strategies could facilitate more individualized and effective clinical management for patients.
